# Surgery of the Face

**Published:** 1878-06

**Authors:** Francis Mason

**Affiliations:** Surgeon and Lecturer on Anatomy at St. Thomas’s Hospital; Hon. Fellow of King’s College, London


### Selections.

### SURGERY OF THE FACE.

BY FRANCIS MASON, F. R. C. S.—SURGEON AND LECTURER ON ANATOMY AT ST. THOMAS'S HOSPITAL; HON. FELLOW OF KING'S COLLEGE, LONDON.

*Abstract of Lecture I.—Part I.—(From the Lancet).*

### DISEASES OF THE FACE.

MR. PRESIDENT AND GENTLEMEN:—My first and most obvious duty is to express to the council my sincere thanks for the honor they have conferred upon me by electing me as the Lettsomian lecturer for the current session. I must confess that whilst I am deeply sensible of the compliment that has been paid me, I am at the same time profoundly conscious of the responsibility that so distinguished a position involves.

In contemplating how I might best occupy the time allotted to the three lectures that I shall have the honor of delivering, I remembered that for many years I had taken considerable interest in the surgery of the face, mouth, throat, and contiguous parts; and, as I had collected several thousand references bearing on these regions—representing, as may be supposed, an immense amount of valuable information—I came to the conclusion that if I sifted some of these references, I might be enabled to submit to the Fellows of the Society many points of more than ordinary or passing interest. I venture, therefore, to engage your attention by describing, as far as my limited time will allow, the surgery of the face, and in bringing this subject before you, I must beg your kind indulgence, inasmuch as I shall necessarily have to refer to many topics with which I feel sure you are all more or less familiar. And I wish to say, at starting, that my object is not to excite sensation, or to provoke controversy by placing before you novelties, but is rather to group together a number of cases I have culled from various sources, including many that have been under my own observation, and which have special reference to the surgery of of a region which from its conspicuousness forms a very important part of the human body.

My first lecture will be devoted to the diseases, the second to injuries, and the third to the deformities of the face.

In order to render my subject as complete as possible, it will be necessary for me to make a few observations on skin diseases, and these need not detain us long.

### ERYTHEMA, ROSEOLA AND URTICARIA.

These diseases are not unfrequently met with on the face, and resemble each other in many particulars.

In *Erythema* the face is covered more or less with blotches of either a bright red or bluish hue. Erythema circinatum is particularly seen on the chin and lips, and appears as distinct circles, or segments of circles. Erythema nodosum is sometimes observed on the forehead, and very much resembles the same disease noticed on the front of the legs in delicate women. It has been mistaken for nodes, but with a little care a correct diagnosis may be arrived at.

In *Roseola* there is less swelling of the skin. The eruption is first of a bright red, which gradually subsides into a deep rosy hue. In this disorder there is more or less redness about the palate and fauces.

*Urticaria* or *Nettle rash* is sometimes limited to the face, and seems to be an aggravated form of erythema or roseola, its characteristic point being the presence of a number of elevations or wheals of variable shape, which are produced by spasm of the muscular fibres of the skin, with a slight effusion of serum. Mr. Erasmus Wilson has pointed out that in the wheals of urticaria an alternate contraction and relaxation of the muscular fibres may be observed, which gives an appearance of puslation, as of an ebb and flow of blood in the capillary vessels.

All these eruptions may, in most instances, be traced to some error in diet, and are prevalent at particular seasons of the year, especially if there be sudden alterations of temperature. I need not add that certain medicines produce similar eruptions. As a rule these diseases require but little treatment beyond attention to diet, with the administration of saline purgatives, alteratives and suitable tonics.

### LICHEN.

Lichen is often found on the face of infants and children, and seems to be due to the irritation caused by teething. The eruption is easily recognized, and if the finger be passed lightly over the skin of the part affected, the cutaneous surface feels like a delicate nutmeg grater.

### HERPES.

Herpes is commonly met with on the face, attacking chiefly the lips, eyebrows and ears. The vesicles, which are dome shaped, appear in groups of patches, more or less circular in form. Moreover they are frequently found to coincide exactly with the cutaneous distribution of certain nerves. Thus, in one case reported, the eruption was limited to the distribution of the left supra-orbital nerve, and throughout showed its distinctive nerve character. Five days before the eruption appeared, there was constant neuralgia in the exact course of this nerve. The vesicles ran upwards over the forehead, and as far as the top of the head, in a longitudinal direction. The eruption is markedly limited to the left side of the forehead and head, the side of the nose, and to the left upper eyelid. Sir James Paget has placed on record an interesting example in which the parts supplied by the second division of the right fifth cerebral nerve were implicated. In this instance, as in the previous one, extreme pain preceded the eruption. This case was, moreover, considered unique n having necrosis of the jaw as a consequence of the intense inflammation of the palate and gums. Twenty-six days from the commencement of the disease one of the bicuspid teeth of the right side of the upper jaw fell out, on the next another, and in a few days later the canine and both incisors. The herpetic eruption was also noticed on the roof of the mouth.

### ECZEMA.

The eyebrows and ears are no uncommon situations for eczema. In this eruption there is a constant serous exudation hence, its title "humid tetter." The viscles have a pointed or acuminated form, and if the disease is unchecked, it assumes a somewhat purulent character, bordering on impetigo, and known as eczema impetiginodes, a disease frequently noticed on the alæ of the nose and on the lips of ill-fed children.

The eruptions of impetigo and ecthyma often coexist, and I need hardly add that their main difference is that in impetigo the eruption is generally confluent, whereas in ecthyma the pustules are solitary, and have an inflamed base.

The constitutional treatment of these diseases must be conducted on general principles, but local applications are particularly suitable when there is much sersous exudation. Ointments of a simple character, such as zinc ointment, or compound subacetate of lead ointment, are especially serviceable, as they prevent evaporation, and thus obviate the production of scabs.

### PSORIASIS AND LEPRA.

These affections are occasionally met with on the face. They are characterised by their dryness, and in this respect differ essentially from eczema. Psoriasis that follows the infecting or true syphilitic sore does not, I venture to believe, commonly affect the face, and when this part is attacked, the inference is, as I think, that the patient has been rather severely poisoned by the disease. From some cause the face and the dorsum of the hands—parts exposed to atmospheric influences and exposed also to observation—seem, fortunately for the sufferer, to possess comparative immunity. The administration of arsenic, iron, iodide of potassium, and the perchloride or other preparations of mercury, generally effect a cure. Locally the white precipitate ointment may be advantageously employed.

### PARASITIC DISEASES.

Of the parasitic diseases we have the type of the animal parasite in *scabies*, which is said by some authorities never to attack the face, but I am sure that it is occasionally found in this region.

The *tineæ* or vegetable parasites are not unfrequently seen on the face. Tinea decalvans manifests itself as bald patches on the skin in the region of the whiskers or beard, and tinea sycosis especially attacks the chin. I may say briefly respecting the treatment, that ointments containing sulphur are invaluable in scabies, and slight mercurial preparations are useful in the different forms of tineæ.

### ACNE.

The only other eruption to which I need now refer is acne, which is perhaps the most common of all diseases of the skin of the face. Acne punctata appears as small black spots, which are the orifices of the sebaceous follicles blocked up with sebaceous matter, dirt and soap. Its most usual situations are those that escape the friction of the towel after washing. In acne simplex there is peri-follicular inflammation, and very often the black spots of acne punctata may be observed at the summit of the small pustules. In acne indurata the inflammatory action is of subacute character. Here the black spot is seldom observable, but instead there is a hard, somewhat diffused lump, which is readily appreciable to the touch. These varieties are often rebellious to treatment, but may be kept in abeyance by the patient attending strictly to diet and by irritating the parts as little as possible. Acne rosacæ seldom appears before the age of forty, and thus differs from the other varieties already described. The face is especially its local habitation. It is observed partly on the nose, extends laterally to the cheeks as a reddish patch, on which a few pustules are here and there scattered. Although it is often attributed by the ignorant to high living, it is very frequently an indication of imperfect digestion, and occurs, as is very well known, in persons of the most abstemious habits.

### BOILS AND CARBUNCLES.

Boils and carbuncles occasionally attack the lips, cheeks and forehead. The more circumscribed swelling in a boil gives it its distinctive character, and it comes, from time to time, in this, as in other parts of the body, in crops, whereas carbuncle is usually solitary, and there is a diffuse, brawny and peculiar hardness due to the arrangement of the soft structures comprising the lips, cheeks and neighboring parts.

With regard to local treatment the question of making incisions seems still to be *sub judice.* It is not often that facial carbuncle is followed by a fatal termination. The fatality of this form of carbuncle is attributed to the susceptibility of the face erysipelas and œdema, and also to the peculiarities of the venous circulation shown by the sudden deaths that have been occasionally noticed after injecting nævi of the face with strong styptics, such as the tincture of the perchloride of iron.

ABSCESSES.

Abscesses of idiopathic origin are not very common, but they are occasionally seen on the face, and the usual variety is that which is known as strumous abscess. It is of slow growth, and exists as a collection of sero-purulent fluid, immediately under a reddened, thin, and generally oblong portion of the skin, the cheek being a common situation. These abscesses are comparatively painless, their progress slow, yet even with the greatest care and attention, they are frequently followed by ugly cicatrices.

Fistulous openings on various parts of the face are not unfrequently met with as the result of decayed teeth, or of necrosis of the subjacent bones, the lower jaw for example. Or they may depend upon the presence of other foreign bodies, as in a case that was under the care of Mr. Henry Smith, which was sent to him in the belief that there was necrosis of the jaw. After a careful examination, Mr. Smith discovered and removed a piece of tobacco pipe, about three inches long which had been embedded in the cheek for several years.*

Sometimes the disease may be traced to the antrum, as in a case that Dr. Richardson kindly placed under my care about three years ago. In operating on the patient I thought it advisable to divide the upper lip in the median line, and having separated the soft parts from the bone I freely opened the antral cavity. A large quantity of offensive cheesy material was evacuated, and the patient made an excellent recovery.

*Assoc. Med. Journal, Nov. 10th, 1854, p. 1017

### ULCERS.

Ulcers of infinite variety are met with in the face, and are frequently the result of injuries by which the soft parts have been more or less damaged; or they may arise idiopathically, as in rodent ulcer and epithelioma, of which I shall speak presently.*

The cachectic ulcer is frequently found on the face. It begins as one or more hard lumps of variable size in the subcutaneous tissue, in which at first there is but little pain.

Whilst on the subject of ulcers, I must not omit to refer to the possible presence of the true infecting syphilitic sore which has been met with on various parts of the face. These sores are important chiefly from a diagnostic point of view, for they have not unfrequently been mistaken for cancer, and have been treated accordingly. In illustration of this point, I may say that in 1872 I saw a man at the hospital, who was twenty-six years of age, and had been operated upon three times within two months for, as he said, cancer of the lower lip. When I saw him he had about his body the clearest evidence of constitutional mischief. What remained of the lower lip presented an uneven, jagged, white patchy appearance, which seemed to me, as well as those who examined him, to be markedly characteristic of syphilitic infection. He was placed on the solution of perchloride of mercury, and he recovered. His object in applying at the hospital was to undergo a further operation.†

### CYSTS.

Cysts, or cystic tumours, of various kinds are frequently found on the face. Perhaps the commonest variety is the sebaceous tumour. Such cysts are of slow growth, and often present at their summit a black spot. They are, as a rule, dome-shaped, and their outline differs in this respect from fatty tumours, which generally present a more flattened form, and are seldom met with in this region.

* See Clinical Lectures by author on Ulcers, *The Lancet*, Sept. 15th, 1877.

† See a paper by the author, on Infecting Sores on the Lips: St. Thomas's Hospital Reports, 1873.

Cystic tumours are usually subcutaneous. Occasionally, however, as in the frontal region, they are submuscular, or subaponeurotic. Sometimes they are met with in connection with the mouth, or even with the cavity of the nose. Their contents vary in character; thus they may either be of cheesy consistence, being principally composed of cholesterine, fat and epithelial scales, or they may be of a more fluid nature.

Hydatid cysts are occasionally met with on the lips and eyelids, and the dermoid cysts are generally situated in the region of the eyebrow and often contain hair.

The best mode, in my opinion, of removing cystic tumours is to transfix them, by which a free opening is at the same time made into the cyst cavity. After the contents of the sac have been squeezed out, the cyst itself becomes more evident, and is easily dissected from the surrounding parts, without much hæmorrhage, provided care be taken to keep the knife close to the sac.

Blood tumours, or hæmatomata, are occasionally seen on the ears, and are said to be frequently met with in insane and idiotic persons.

(TO BE CONTINUED.)

